# Examining the Potential of Social Robots to Increase Adherence in Internet-based CBT

**DOI:** 10.1007/s12369-026-01417-8

**Published:** 2026-07-13

**Authors:** Elly A. Konijn, Gidon Peeper, Tijs Portegies, Nadia Garnefski, Vivian Kraaij, Sascha Struijs

**Affiliations:** 1 School of Social Sciences, Department of Communication Science, Media Psychology Program Amsterdam, Vrije Universiteit Amsterdam, Amsterdam, The Netherlands; 2 Faculty of Sciences, Department of Computer Sciences, University of Amsterdam, Amsterdam, The Netherlands; 3 Rotterdam University of Applied Sciences, Creating010, Rotterdam, The Netherlands; 4 Faculty of Social and Behavioural Sciences, Department of Clinical Psychology, Leiden University, Leiden, The Netherlands; 5 Faculty of Behavioural and Movement Sciences, Department of Clinical, Neuro and Developmental Psychology, Vrije Universiteit Amsterdam, Amsterdam, The Netherlands

**Keywords:** Unguided iCBT, e-Health, Adherence, Avatars, Social robots, Therapeutic alliance

## Abstract

Internet-based applications of Cognitive Behavioral Therapy (iCBT) are promising to alleviate mood problems and depression. However, versions without personal support (i.e., unguided) still fall short in establishing meaningful therapeutic relationships, despite recent versions also embedding virtual avatars, potentially resulting in low levels of adherence. The current paper systematically examined whether a physically present embodied social robot, automatically operating (i.e., no WOz), may yield stronger therapeutic alliance and increase adherence compared to screen-based therapeutic interventions through experimental research designs. In two separate experiments, participants were randomly assigned to either a robot intervention or screen-based control condition with or without an avatar, addressing mild mood issues in three subsequent intervention sessions. Overall, results showed the social robot intervention to increase therapeutic alliance, adherence, and satisfaction compared to the same screen-based intervention, with alliance in a mediating role, but did not significantly improve participants’ mood. Future research is warranted with larger samples suffering mental health issues. The studies highlight the potential of social robots to act as guidance in unguided iCBT interventions and enhancing its effectiveness.

**Supplementary Information:**

The online version contains supplementary material available at https://doi.org/10.1007/s12369-026-01417-8.

## Introduction

A significant proportion of the human population is affected by depression and mood issues, for example, 13–25% of adolescents in Europe [1]. This has increased by the Covid-19 pandemic, while facing a shortage of mental health professionals and a lack of financial resources [2, 3]. This causes long waiting lists and further worsens health issues for many. Cognitive Behavioral Therapy has proven to be an effective method for a broad range of mental health problems, offline or online, and with or without the guidance of a therapist [2, 4]. An important difference between guided and unguided therapy is the presence or absence, respectively, of a mental health or trained professional. However promising, unguided versions of iCBT do suffer from low adherence rates [5]. Hence, it is urgently needed to further examine the potential of new communication technologies. A social robot is such a new communication technology that can be examined in its potential to increase adherence in unguided therapy.

Despite considerable research showing the promising potential of internet-based CBT to alleviate mood problems and depression among adolescents and (young) adults [6–8], it still falls short in establishing meaningful therapeutic relationships (i.e., therapeutic alliance). Even though recent versions embedded virtual avatars, adherence rates are still rather low [9, 10]. The absence of a therapist in unguided iCBT lacks the possibility for therapeutic alliance, which was found to play an important role in CBT’s overall efficacy [4, 10] and lowers adherence which makes participants quit the program early [11]. We argue that the physical embodiment of a social robot may stimulate such therapeutic alliance and increase adherence among people seeking support for their mental health.

Even though still in its infancy, research on social robots thus far suggests that robots easily elicit feelings of connectedness [12–14]. Such connectedness might be translated into therapeutic alliance when implemented in a therapeutic context. Therefore, for the current study, we argued that a physically present, embodied social robot would bring stronger feelings of connectedness or alliance which may then also improve adherence, compared to a screen-based therapeutic intervention. Because this research applies CBT with social robots for the first time, we focused on mild mood issues (cf. sub-threshold depression) among student populations.

### Guided and Unguided iCBT, Alliance and Adherence

To alleviate the workload of psychologists and psychiatrists who cannot keep up with the substantial demand for (professional) help, online possibilities for people who seek help have emerged to complement common traditional offline therapy and counseling [12, 15, 16]. Internet-based CBT is based on the same principles as CBT [17], with the main difference that it can be administered online and without the presence of a therapist (i.e., unguided; [4]). The therapy can be followed on a mobile phone or through a computer program at any convenient place, instead of in the room of the therapist. Considering this improvement logistically, as well as financially, and the possibility to alleviate the stigma because iCBT can be followed anonymously, it is a promising alternative to face-to-face CBT.

Research shows that internet-based CBT is generally effective in the treatment of mental disorders, and in some cases its effects are comparable to those of face-to-face CBT [18]. Guidance typically consists of regular feedback or coaching by trained professionals added to the internet version [19, 20]. Concerns have been raised about the *un*guided form of therapy as it helps significantly less than guided iCBT or face-to-face CBT [5, 8, 21]. Concerns about unguided iCBT have traditionally included relatively small effect sizes, lower adherence, and higher dropout rates [11, 22, 23]. At the same time, unguided interventions are scalable at low cost, making them particularly relevant for prevention at the population level.

Research showed that the frequency and intensity of guidance played a significant role in the effectiveness of the therapy, that is, with more personal guidance, better results. Although *un*guided iCBT can contribute to significant improvements in one’s mental health [11], findings show a relatively high drop-out rate of unguided iCBT users when compared to users of the guided or face-to-face alternatives [8, 11]. Drop-out rates, also called non-adherence, appear to be twice as high in the unguided version of iCBT ([5, 24–26]). The low adherence rate of unguided therapy is considered an obstacle to its potential success [4] and is defined as the main challenge of unguided iCBT in a meta-analysis [11]. Although the individual's freedom seems to relieve accessibility issues, it comes with a certain risk. In unguided iCBT, usage of the intervention is largely at the discretion of the user. Compliance is entirely voluntarily, which makes the decision to quit the program relatively easy.

More recent studies show a more nuanced picture: Differences between guided and unguided interventions may be smaller than previously assumed, and outcomes can depend on factors such as symptom severity, population characteristics, or the nature of support provided [7, 27]. Some recent trials even suggest that adding human guidance does not necessarily increase effectiveness, though it may enhance adherence [28].

Importantly, in guided iCBT, the level of alliance between patient and therapist shows to be highly associated with the outcomes of the therapy [10]. The lack of a therapist in unguided iCBT has often been reported as the reason for low adherence and the disappointing efficacy in general: the impersonality of non-guided iCBT. However, in most research that examined the adherence rate of unguided iCBT, the object of study was primarily an intervention through text-based websites (Provoost et al., 2017; 2021). To overcome this problem, several (commercial) chatbots or avatars have been developed [29, 30]. These avatars in iCBT are one of the current workarounds to deal with the shortage of therapists and suggest some sort of guidance.

### Newer Forms of iCBT: Avatars and Social Robots

The use of social avatars have showed to be an effective way to assist with providing therapy [31–33]. A scoping review showed that these social avatars can be used for a variety of mental health issues (Provoost et al., 2017). The use of avatars within Internet interventions also showed higher levels of alliance [34] compared to a text-only option [35]. More specifically, Heim et al., [35] were among the first to make a distinction between different unguided therapy facilitators and found that people then report higher feelings of alliance, because avatars simulate human behavior more closely than, for example, a text only alternative (e.g., self-help books). Thus, not only can an alliance be formed with an online facilitator that ‘replaces’ or attempts to approximate the mental health therapist, but the level of experienced alliance also differs between individual unguided iCBT facilitators.

There are, however, few social avatars that are used specifically to provide iCBT [36]. An example of an avatar used to provide iCBT-based conversations is the Woebot [32]. The Woebot is a robot-like cartoon chatbot created to help its users with mental health issues, which was found to be significantly more engaging than the text-only option. Although the study did not measure the level of adherence and the effect of the avatar used in the application, the results suggest a higher drop-out rate in the text-only intervention. Other studies have similarly suggested that participants use the social avatar intervention more than the text-only variant, and that the higher engagement level of the social avatar is due to its virtual embodiment [25, 37, 38].

Nowadays, in many unguided online therapies, an avatar is used to create at least some form of humanity in an otherwise solely artificial environment. And even though the use of an AI-based therapy with an avatar is already accepted by patients [9], it is again significantly more effective when a human therapist is involved. Provoost and colleagues [25] explain, moreover, that a higher level of engagement is linked to a significantly higher level of adherence. However, studies on social avatars providing iCBT are scarce, and while scholarship is emerging about this topic [23], more research with a focus on the exact role of the virtual embodiment of the social avatar – rather than the conversation it provides – is needed.

In addition to the use of social avatars in mental healthcare, another new communication technology is used: a social robot in the form of a Robo-therapist [39]. As this field is quite new, much is still unknown about social robots providing iCBT [16, 40]. Studies on the effects of social robots in healthcare are positive, despite methodological shortcomings such as lacking adequate comparison studies, and overall suggest that embodied robots easily elicit feelings of connectedness or a socio-affective bond [12, 13, 16, 41, 42]. Furthermore, instructions from an embodied robot were followed more closely than from a computer tablet with the same software and voice [14]. Various studies report on the additional and positive role of a robot’s physical embodiment and presence in the same physical space in enhancing social interaction, such as the perceived hedonic quality [43], social perception and overall evaluation of the interaction [44]. Also in the field of education, research showed the added value of a robot’s embodiment [28, 45].

A first study that compared a social robot with an avatar in a mental healthcare setting, yielded important insights: The physical embodiment of the robot enhanced the social responses (verbal and non-verbal) of the participants because of its social presence [46]. Lee et al., [46] concluded that the physical embodiment of the social robot is not just an additional benefit, but an ‘essential dimension’ when it comes to social interaction. In line, Jeong et al., [41] explain the uniqueness of a social robot’s embodiment in its successful creation of a working alliance with the user, and found increases in students’ psychological well-being, mood, and motivation to change following the robotic intervention.

Another study provided iCBT to older adults suffering from depression [47] and found that robot-based therapy can be a valuable alternative to the traditional face-to-face therapy because of the experienced comfort and engagement while working with the social robot. Other studies using a social robot for other types of therapy (i.e., not iCBT) reported relatively high levels of enjoyment and highlight the robot’s unique ability to provide live social support due to its physical presence [48, 49]. In reviewing 38 individual studies, Li [50] concluded that physical embodiment was experienced more positively and resulted in a higher level of social responses than the virtual embodiment of social avatars. The current research builds on that understanding by bringing a social robot into a comparative analysis as an alternative for a mental health therapist that resembles human interaction more closely and might enhance the adherence for therapy [51].

Following from the above, both the social avatar and the social robot would exert higher levels of engagement compared to a text-only option ([46, 50], which can result in higher levels of adherence [25]. Direct comparisons of the different iCBT facilitators – text-based website, virtual avatar, and embodied social robot – on adherence has to our knowledge not yet been examined systematically through an experimental research design. Following the line of thought of the studies mentioned above, we hypothesized for the current research that higher levels of embodiment in new communication technology would increase alliance and adherence. Thus, compared to a text-based website (a), participants who follow unguided iCBT provided by a virtually embodied social avatar (b) or a physically embodied social robot (c) were expected to report higher levels of adherence (H1) and alliance (H2).

Furthermore, we examined whether feelings of alliance can possibly explain a higher level of adherence by a social avatar or social robot intervention. Alliance appears to be crucial in therapy as earlier research highlights that the formation of an alliance with the therapist enhances the levels of adherence [52, 53]. A physically embodied social robot might specifically be tailored to bring such therapeutic alliance in unguided iCBT following the above reported research on social robots ([12, 14, 42]). Hence, we will test whether alliance mediates an iCBT facilitator’s effect on adherence (H3). To test our hypotheses, we conducted two experiments that built upon each other in terms of materials and procedure.

## Study 1

The aim of this study was to contribute to the understanding of the potential of a social robot in unguided therapy. In order to test whether increasing levels of embodiment of unguided therapy facilitators affect users’ feelings of alliance and adherence, we compared a text-based website without embodiment with the same text-based website including a social avatar, thus a virtual embodiment in the form of an avatar. These screen-based versions were each also compared with a social robot that was physically embodied. We took care that the intervention in each condition was as much similar as possible. The experimental comparison focused on the users’ level of adherence to the therapy in response to interacting with either one of the iCBT facilitators as well as on the degree to which users experienced therapeutic alliance. In addition to participants’ adherence and alliance, only in Study 1, the reported level of loneliness was also measured because Study 1 was conducted during the COVID-19 pandemic (Study 2 thereafter).

### Method

#### Design and Participants

An experimental mixed between-within design was employed to test whether adherence and alliance differed among three types of unguided iCBT facilitators, varying in levels of embodiment: (a) a text-based website; (b) a social avatar (i.e., virtual embodiment, 2D);and (c) a social robot (i.e., physically embodied, 3D). Dependent variables were adherence and alliance. To measure adherence, three subsequent sessions were created, per condition, following standard iCBT procedures, thus, including an introduction and exercises (see below). The feeling of alliance was measured afterwards and also examined as a possible mediator between the iCBT facilitator andadherence. Loneliness was included as a covariate, a possible moderator, in Study 1. Finally, age and gender were included as control variables. The study was approved by the Institutional Ethical Review Board.

Participants were 39 students (age range 19–29 years;M= 23.33;SD= 2.34; 61.5% female) who were randomly assigned to one of eachcondition (i.e., either the text-only website, the avatar, or the social robot). During the interaction, exercises were provided following thesession. Interventions took place on three separate occasions within a week’s time. A G*Power analysis was performed, calculating therequired sample size (cf. MANOVA, repeated measures, between factors analysis) resulting inN= 36 participants to test the hypotheses ata significance level of 0.05 and a power of 0.8 [54] with an estimated effect size of 0.4. Four participants dropped out during theexperiment (i.e., leavingN= 39). These four drop-out cases all occurred in the text-only condition.

### Procedure

The study was presented as an experiment for students that experienced feelings of loneliness during the COVID-19 pandemic, recruited through the first authors’ university as a convenience sample. Students applied online by filling in a starting date, which was marked as day one of the experiment. On day one, day three, and day five of the experiment, all participants received an email with a link that led to the session of that day. For privacy reasons and following ethical guidelines for research (including active consent), the participants were asked to create their own participation number, which was not to be communicated with the researcher so that their answers could not be traced back to them. Participants could log on to each session by using the same participation number as the first time. Participants in each condition were instructed to speak answers to questions out loud for technical reasons. This was needed due to limited speech recognition technology of the NAO robot. Furthermore, it was emphasized that the answers were not saved after the session.

**Fig. 1:**
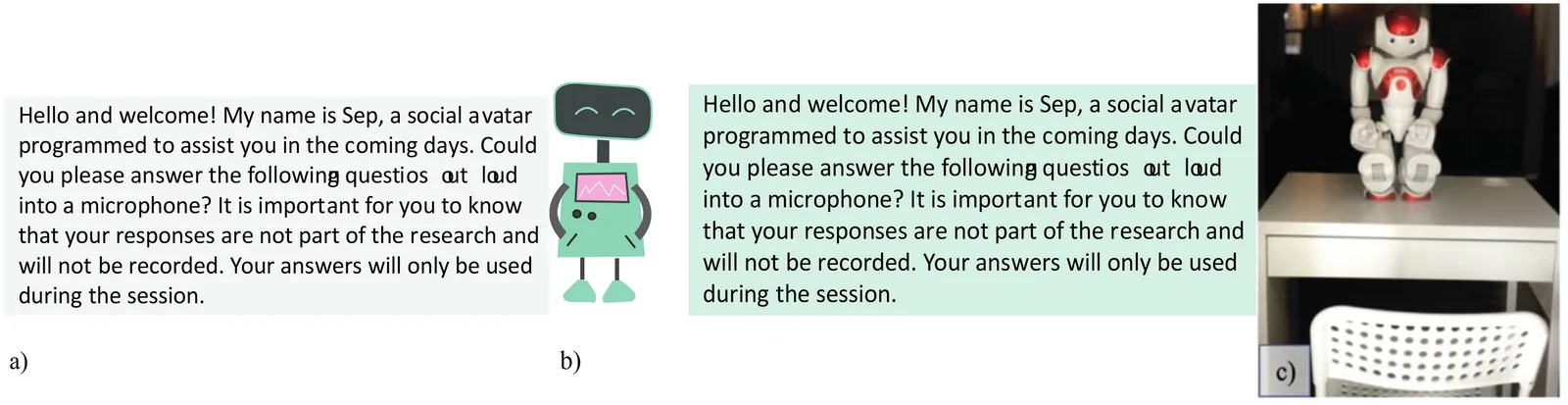
Therapeutic facilitators in unguided treatment used in Study 1. Note. The three types of therapeutic facilitators compared in the current study: (a) text-only; (b) virtual avatar; (c) social robot

The procedure for the three conditions was as similar as possible, yet differed in that the participants who were assigned to the social robot condition received an email with the location of the social robot and asked to meet the robot there. The robot was positioned on a table and connected to a computer (Fig. 1). The participant was instructed to push the play button upon arriving at the designated location – a neutral waiting area. When the participant clicked on the play button, the social robot started the session. At this location, the researcher was not in eyesight as this could influence the participant. The social robot was at the same place for each of the three repeated sessions (T1-T3) within the robot condition (no robot was visible in the other conditions). After the session was finished, the participants were asked, by the social robot, avatar, or in text, to fill out a questionnaire.

In the first introductory session, participants were asked to present themselves and share how they would reduce feelings of loneliness. Thereafter, participants were asked to keep a diary in the next two days, briefly analyzing their own thoughts such that each time a distressing thought came up, to ask themselves if it was a fact or an opinion [55]. It was emphasized that the diary was strictly personal and answers would not be shared. Finally, participants were asked to complete a questionnaire. In the second session, participants were asked to reflect on their diary entries and report how often they had written something. It was emphasized that there were no right or wrong answers. Then, the therapy facilitator introduced a breathing exercise with the instruction to breathe in for seven seconds and to breathe out for ten seconds [55]. In closing the second session, the participant was instructed to repeat the breathing exercise whenever a distressing thought came to mind in the following days. In the third and last session, participants were asked to reflect on the breathing exercise and to report how often they had performed the exercise. Then, the participants were asked to write down a distressing thought, followed by a breathing exercise. Thereafter, the unguided therapy facilitator asked whether the thought that the participant had written down was a fact or an opinion. Finally, the participant was thanked and asked to complete the final questionnaire.

### Materials

The materials created to examine the differences between the three types of unguided facilitators were each based on the same content following standard practices in iCBT [55]. See visualizations in Fig. 1.

#### Text-Based Website

For the text-based website, the materials for the treatment were integrated in a standard software environment for questionnaires (i.e., Qualtrics). By embedding the materials in the questionnaire, participants did not have to switch programs when answering the questions. The stimuli resembled a commonly used text-based website to perform unguided iCBT (Karyotaki et al., 2018).

#### Social Avatar Website

As the use of a social avatar in mental health practices provided good outcomes [23, 32], we chose for a similar design for the social avatar in our study. The social avatar and the text-based website differed only in the presence or absence, respectively, of a virtually embodied entity (see Fig. 1).

#### Social Robot

For the social robot condition, a NAO robot was programmed to follow the same treatment as in the other conditions. Different from the two other conditions, the NAO robot was physically present while performing the therapeutic modules. The NAO robot did not make any movements during the sessions, as the other two stimuli also could not move. We kept the conditions as similar as possible, except for the type of embodiment. Choregraphe V2.6.8 was used to program the NAO robot. Data and materials are available on OSF.

### Measures

#### Adherence

Adherence is defined as the “extent to which individuals are exposed to the content” of the treatment [4], measured through the (ir)regularity of attendance to therapy. In the current study, we extended this operationalization by including participants’ exercise execution as an indicator of treatment exposure. This approach recognizes that participants may perform the exercises not only during the sessions but also in their daily life in between sessions. To assess this, two self-help exercises (see *Procedure*) were included in the sessions of the treatment. At the second and third session, the participants reported the extent to which they performed these exercises during the preceding days, with response options ranging from “1 = totally disagree” to “6 = totally agree” (Spearman-Brown = 0.84, *M* = 2.94, *SD* = 1.58, *N* = 39). The total score for adherence was calculated as the average across the two self-help exercises, with higher scores indicating stronger adherence.

#### Alliance

Alliance is here understood as how much participants felt involved with the intervention or therapeutic facilitator (cf. [4, 46]. To measure alliance, ten items were adapted from the *perceived other’s co-presence* scale [56] with items like “My interaction partner was intensely occupied in our interaction.” To counterbalance, four items measured how distant participants felt from the facilitator (e.g., “The facilitator created a feeling of separation.”) that were recoded before analysis. Answering options ranged from “1 = totally disagree” to “6 = totally agree” (Cronbach’s α = 0.91, *M* = 3.98, *SD* = 1.13, *N* = 35). The feeling of alliance was only measured once, after all sessions were completed, with a higher score indicating stronger alliance.

#### Loneliness

Even though the scope of this paper was not on mood optimization, it was informative to include loneliness when testing our hypotheses. The level of loneliness was measured twice, once before the three interaction sessions and once after. The loneliness short scale by De Jong Gierveld and Van Tilburg [57] was used, including six items, measuring both emotional loneliness (e.g., “I feel often left alone”) and social loneliness (e.g., “There are plenty of people I can rely on in case of emergency.”; to be recoded). The answering options to each item ranged from 1 = no, 2 = now-and-then, to 3 = yes. Following instructions [57] all answers with ‘now-and-then’ and ‘yes’ were coded as ‘1’ (indicating loneliness), and all answers with ‘no’ were coded as 0. All points were summed to arrive at a loneliness score (max = 6) with a higher score indicating a higher level of loneliness (i.e., measured at ordinal level and thus no reliability measure).

### Results

#### Preliminary Analyses

 To track down possible confounds, we correlated gender and age with adherence to exercise 1 and 2 as well as to alliance (see Suppl. Mats., Table S1). The only significant correlation (at the 0.05 level) was between gender and alliance (*r* = 0.38, *p* = 0.027) with female participants reporting higher feelings of alliance.

#### Testing Hypotheses

**Fig. 2:**
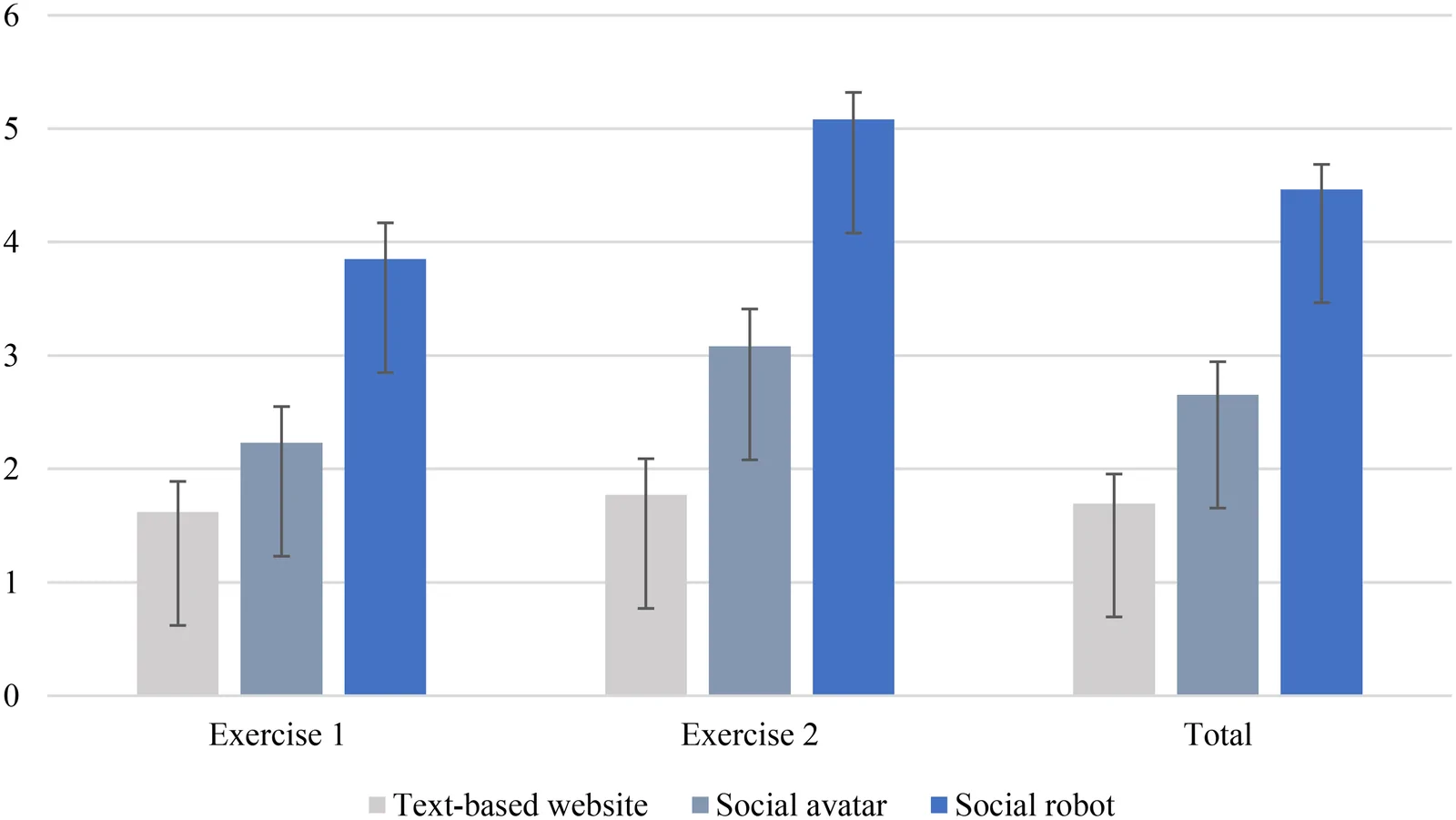
Mean levels of adherence to the exercises in between sessions

To test H1, results of a one-way MANOVA for the type of unguided facilitator on adherence to Exercise 1 and to Exercise 2 combined, showed significant differences (*V* = 0.66, *F*_(4,72)_ = 8.92, *p* = 0.000, *η*_*p*_^*2*^ = 0.33), indicating an overall significant effect of condition on the total level of reported adherence (Fig. 2). The between-subjects effects showed significant differences between the unguided facilitators on adherence for both exercises (*F*_(2,36)_ = 14.42, *p* = 0.000, *η*_*p*_^*2*^ = 0.45). That is, for Exercise 1, participants using the social robot reported significantly more adherence (*M* = 3.85, *SD* = 1.14) than participants in the text-based condition (*M* = 1.62, *SD* = 0.96) (*t*_(24)_ = 5.39, *p* = 0.000), and significantly more than in the avatar condition (*M* = 2.23, *SD* = 1.17) (*t*_(24)_ = 3.57, *p* = 0.001). No significant differences were found in the level of adherence to Exercise 1 between the participants in the text-based and the avatar condition.

Between-subjects effects also showed significant differences between the three unguided facilitators on adherence to Exercise 2 (*F*_(2,36)_ = 30.81, *p* = 0.000, *η*_*p*_^*2*^ = 0.63). Participants following unguided therapy provided by the social robot (*M* = 5.08, *SD* = 0.86) reported significantly more adherence for Exercise 2 compared to the participants in the text-based website condition (*M* = 1.77, *SD* = 1.17) (*t*_(24)_ = 8.23, *p* = 0.000), and the avatar condition (*M* = 3.08, *SD* = 1.19) (*t*_(24)_ = 4.91, *p* = 0.000). Participants in the avatar condition reported significantly higher levels of adherence in Exercise 2 compared to participants in the text-based condition (*t*_(24)_ = 2.83, *p* = 0.009).

To test H2, results of the one-way ANOVA for the differences between the unguided facilitators on alliance (*n* = 35) showed significant effects (*F*_(2,35)_ = 11.58, *p* = 0.000, *η*_*p*_^*2*^ = 0.42). Independent-samples *t*-tests (two-tailed) revealed that the social robot (*M* = 4.91, *SD* = 0.35) yielded significantly higher levels of alliance compared to both the text-based website (*M* = 3.24, *SD* = 1.17) (*t*_(20)_ = 4.88, *p* = 0.000) and to the social avatar (*M* = 3.57, *SD* = 1.03) (*t*_(24)_ = 4.43, *p* = 0.000). The text-based website and the social avatar showed no significant differences in the reported level of alliance.

Thus, according to these results, participants following treatment provided by the physically embodied social robot (c) reported higher levels of adherence in comparison to the participants who followed regular text-based treatment (a) or with a virtually embodied social avatar (b). Only over time (after the second exercise) did the social avatar yield better results than the text-only website. H1 is thus supported with participants in the robot condition showing the highest level of adherence. The results further support H2 in that the participants interacting with the social robot reported more intensely experienced feelings of alliance than either the virtual avatar version or text-based website.

#### Testing Mediation of Alliance

In following Baron and Kenny [58] to conduct mediation analysis, the result of a linear regression of alliance as predictor on the total score of adherence was significant (*R*^*2*^ = 0.49; *F*_(1,34)_ = 32.11, *p* = 0.000). With more alliance, adherence increased (standardized *β* = 0.82, *t* = 5.67, *p* = 0.000). As a next step, we tested whether there was an indirect effect of type of the unguided facilitator on the level of overall adherence through alliance (H3). Therefore, we performed Hayes’ PROCESS macro (model 4) for mediation [59]. In following instructions, we regarded the unguided facilitator as the multi-categorical independent variable, the feeling of alliance as the mediator, and adherence as the dependent variable. Because only two levels can be included as independent factors in PROCESS, the full mediation analysis occurred in three steps as reported in the Suppl. Mats. Here, we only report the mediation for the comparison of most interest in the current study.

**Fig. 3:**
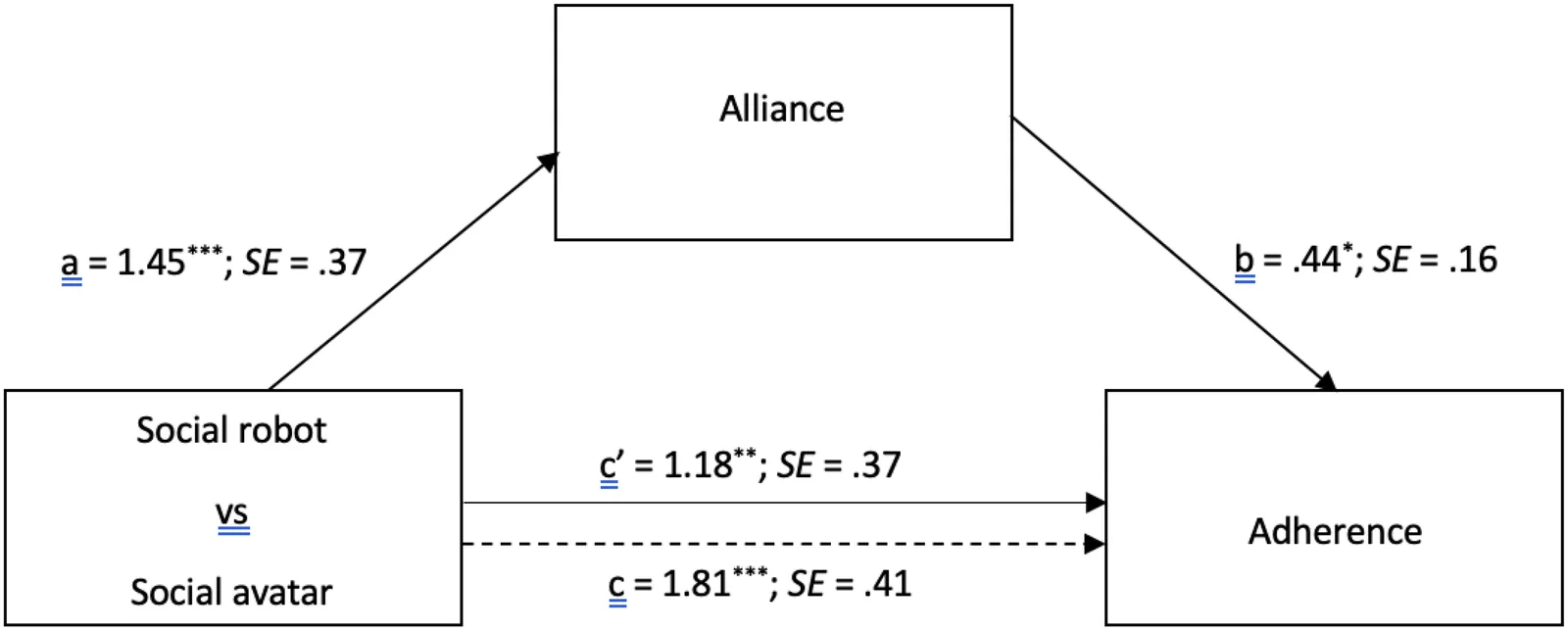
Mediation analysis (social avatar vs. social robot). Note. Mediation analysis predicting indirect effects of the type of unguided facilitator (social robot vs. social avatar) on Adherence through Alliance. Unstandardized path coefficients, * *p* < .05, ** *p* < .01, *** *p* < .001

In accordance with the analysis of variance above, the social robot evoked a significantly higher level of adherence compared to the avatar and showed a significantly stronger relationship for adherence (*B* = 1.18, *p* = 0.007). The social robot was also significantly stronger related to alliance than the avatar (*B* = 1.45, *p* < 0.001). Higher levels of alliance further resulted into higher adherence (*B* = 0.44, *p* = 0.011). The indirect path from the type of unguided facilitator on adherence via alliance showed to be significantly different from zero (point estimate = 0.63, 95% *CI* [0.18, 1.23]). Moreover, a Sobel test on the direct and indirect path significantly differed (*z =* 2.25, *p* = 0.025). Thus, the difference between the robot and the avatar on Adherence was mediated by participants’ feelings of Alliance as illustrated in Fig. 3.

In conclusion, H3 was supported by the main results, showing that adherence for the robot condition was mediated by alliance when compared to the avatar condition, as well as compared to the text-only (Suppl. Mats., Fig. S3). Alliance did not mediate adherence for the text-only or avatar as therapeutic facilitator.

#### Exploratory: Can iCBT via Robots Reduce Loneliness?

With respect to the different moments in time, the average sum score of loneliness at the beginning of the experiment (*M* = 3.41; *SD* = 1.69) was higher compared to after the experiment (*M* = 2.97; *SD* = 1.63; max score = 6). However, the loneliness scores between the different conditions (details in Suppl. Mats., Table S2) did not significantly differ before (*F*_(2,34)_ = 0.046, *p* = 0.955) and after the experiment (*F*_(2,32)_ = 0.45, *p* = 0.639).

#### Discussion Study 1

In this first study, we compared three variations of the same therapeutic intervention to test whether increased levels of embodiment of the therapeutic facilitator in unguided interventions would increase its effectives. Overall, results supported our hypotheses that both therapy adherence and therapeutic alliance improved with increased physical embodiment. In comparing a text-only website with a similar website including a social avatar (i.e., virtual embodiment), and a physically embodied social robot in an experimental design, main results showed that participants in the social robot condition reported both significantly higher levels of adherence and alliance compared to both the text-only and the social avatar condition. Alliance was also found to mediate adherence in the social robot intervention compared to the other two types of unguided facilitators.

Whereas the results generally were in line with previous findings for adherence and brought new insights regarding therapeutic alliance, several shortcomings warranted a follow-up study. First, the measure of adherence in our study differed from common interpretations in iCBT research. We measured the degree to which people executed their exercises, in between sessions, as part of the treatment, rather than their attendance to the treatment sessions [4]. Likewise, our measure for therapeutic alliance was different than what is more commonly understood as therapeutic alliance [33] because we used a measure for ‘social presence’ [50]. Therefore, in Study 2, we used more applicable measures for these dependent variables.

Second, Study 1 relied merely on common self-help guidance [55], for practical reasons, whereas a next study could apply an intervention more closely resembling current iCBT practices. Therefore, Study 2 translated common modules in iCBT into avatar and robot versions as therapeutic facilitators to gain more insight into most suitable alternatives for providing unguided iCBT, without the presence of a human therapist.

Another improvement for Study 2 resides in the location to attend the sessions. Participants assigned to the text-based and avatar conditions, both screen-based, followed the sessions from a secure location of their choosing, as long as they had the ability to connect to the Internet. By contrast, the participants in the social robot condition had to physically go out to attend the therapy session. Thus, the amount of effort participants had to invest in the various experimental conditions differed. While this difference might have influenced the results, it should also be noted that this might actually be representative of real-life situations when people attend face-to-face therapy sessions. It might well be that the investment of time and effort of going to a therapy session already contributes to its success and may further explain the high attrition rates in screen-based iCBT followed from home. Therefore, in Study 2, it is important to compare sessions with a social robot versus a screen-based avatar both at a remote location where the participants have to go to.

## Study 2

Given the promising results of our first study regarding the potential of a social robot to take on the role of iCBT facilitator, Study 2 was designed to significantly improve the design, materials, and measurements in a more controlled laboratory setting. We did not include a text-only version, because of its clear underperformance in Study 1. In Study 2, we compared the same iCBT intervention offered by either a screen-based avatar or a physically embodied robot. We collaborated with Caring Universities to more closely resemble current iCBT modules than in Study 1. Caring Universities (https://caring-universities.com/) is a joined initiative from several universities (also including applied sciences) in the Netherlands to offer multiple free online guided co-created e-health programs, designed by professionals, to improve mental wellbeing of their students [60]. One of those programs is MoodPep [61], on which the current intervention is based, and is a form of iCBT aiming to improve the mood of its users by addressing daily struggles, starting new activities, and learning to cope with negative thoughts, among others. MoodPep offers seven modules to university students to improve their well-being, to be completed on a weekly basis. These modules include content and tasks that reach from psychoeducation to exercises. Sometimes it also requires the user’s input. Three adapted modules have been implemented in our design (see ‘materials’).

Furthermore, all interventions took place in a laboratory setting at the university, thus, equating conditions in that all participants had to come to the lab for the sessions. Also in other respects, we made the appearance features of the avatar and robot conditions more similar than in Study 1 (see Method). All measures have been improved in using measures that are more common in this field to measure adherence and alliance (see Measures). Finally, rather than including loneliness, we added measures for mood or mental health in general and satisfaction with the intervention or therapy, which are more common in iCBT research ([2]; van Ballegooien et al., 2014; Provoost et al., 2021).

Theoretically, we followed the same arguments and hypotheses as in Study 1, with two additional hypotheses due to the two added dependent variables (i.e., mood and satisfaction), following the same logic as for adherence and alliance. Hence, we expected positive effects of the robot condition compared to the avatar for each dependent variable.

### Method

#### Design and Participants

**Fig. 4:**
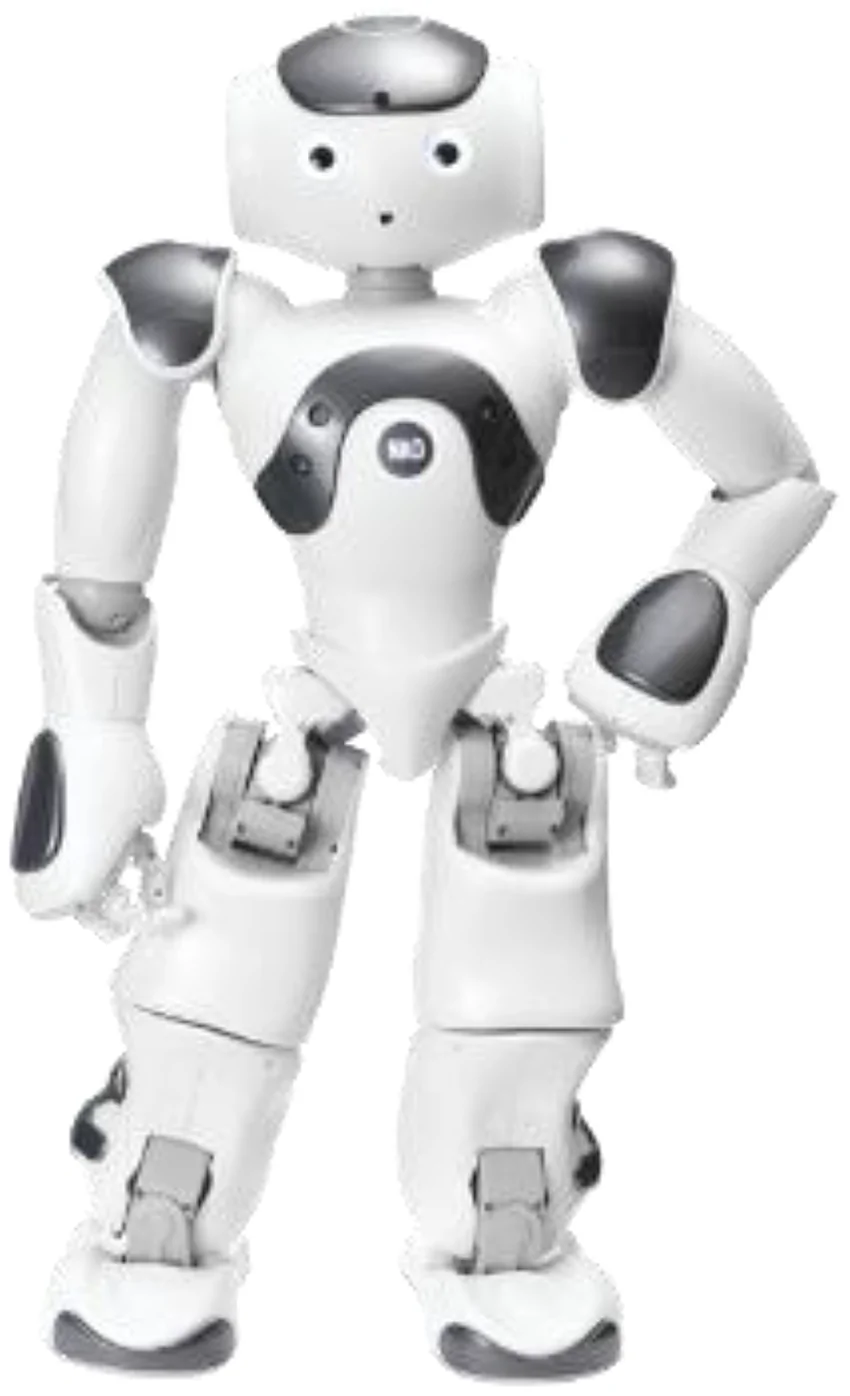
Picture of Nao robot as avatar

**Fig. 5:**
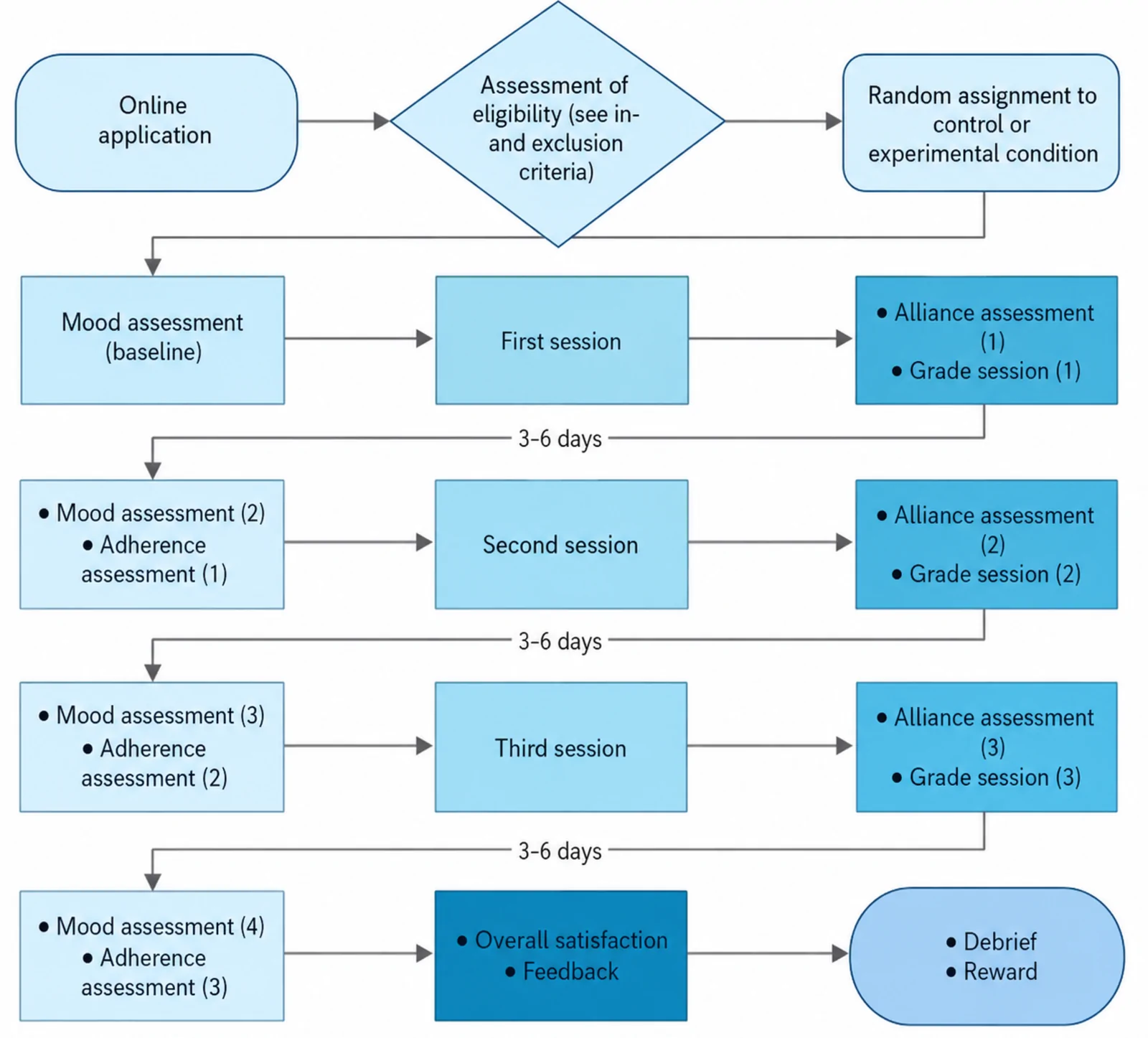
Flowchart of the procedure of study 2

We tested our hypotheses in a mixed 2 (iCBT facilitator: robot vs. avatar) x 3 (sessions) between-within subjects experiment with repeated measures over time during three sessions for adherence, alliance, mood, and satisfaction with the session. The screen-based avatar condition used the very same intervention as the robot condition and we used a picture of the same NAO-robot to represent an avatar (see Fig. 4). Hence, the screen-based version was as similar as possible to the robot condition and followed current principles as applied in unguided iCBT. Mood was measured 4 times, also including a posthoc measure 3–6 days after all sessions were completed. Overall Satisfaction was measured once 3–6 days after all sessions were completed (see flowchart Fig. 5).

Participants (*N* = 22, *M-*age = 20.9, *SD-*age = 2.4) were randomly assigned to either the experimental robot intervention or screen-based control condition with a laptop. Following the same G*Power analysis as in Study 1, yet for 2 groups, resulted in *N* = 42. The assignment of participants to conditions as well as the planning for sessions was automatized with 3–6 days in between sessions, which could be scheduled by the participants themselves. The study was approved by the Institutional Research Ethics Review Committee (RERC, registration number 2023-4-13-654) and all participants signed an informed consent form.

### Materials

The intervention was based on MoodPep (cf. Caring Universities; Cuijpers, Aurbach, et al., 2019; [61]) from which three iCBT modules were selected that we considered most suitable for the present study and to be implemented in a robot. We also included the exercises in between sessions. Each module had an interaction duration of 30 min and was similar to the current MoodPep procedure, yet, without feedback from a coach of Caring Universities. In close cooperation with MoodPep’s developers [61], three of the seven original modules were selected to function as the basis for the intervention of this study. This selection was made to bring down the total number of sessions to make the experiment more feasible. The three modules targeted ‘activity stimulation’, ‘replacing negative with positive thoughts’, and ‘setting personal goals’ and were presented in this order.

The first session was named “Breaking the vicious circle”, and was developed to stimulate activity by providing the participant with (and elaborating on) a broad list of positive activities. The second session supported the user in replacing negative thoughts or feelings with positive ones, hence named “Evoke a positive feeling”. Before evoking a positive feeling, participants were asked to think about a negative thought or event. The third and final session “Finding personal goals” helps the user with creating feasible and concrete goals, for example, by distinguishing them from intangible wishes.

In both conditions, participants were asked to write down their answers to questions in interacting with the iCBT facilitator. The form was for them to take home, so they could remember as much as possible from the therapy and also apply it outside of the lab. Each page showed a picture of the NAO robot (Fig. 4), which was referred to as ‘Noa’ - a unisex name and similar in sound to NAO.

In the experimental condition (*n* = 12), the NAO robot was programmed to automatically operate in providing MoodPep verbally, introducing itself with the name Noa. While talking, the robot was programmed to perform subtle, human-like movements to make it appear more lifelike. The participants were asked to verbally repeat the answers they wrote down as a form of interaction, to resemble a dialogue. Participants were instructed to grab the hand of the robot (that has sensors) to indicate the end of their verbal response, after which the robot continued with the intervention.

The control condition resembled the current standard of care: unguided iCBT with an avatar. In the control condition (*n* = 10), participants completed the MoodPep modules on a laptop with an avatar instead of a robot, following the very same protocol in the lab. The control condition was created on WordPress after the original of Caring Universities. Some parts of the therapy (such as lists of activities or pictures that were shown on the monitor) were also printed on a form (a new form for each session).

### Measures

All Likert-type measures were completed via an online form on Qualtrics as described below.

#### Adherence

Adherence was assessed in two ways, both through session measures and drop-out rates as is common in iCBT research [4, 11]. Adherence to the therapy was measured each session by asking participants three questions about how often they thought about tips, or engaged in activities that were mentioned in the therapy, based on earlier research [11, 25]. An example question is: “How many times in the last week did you think about something that we talked about?”. Answer options were provided through rating scales: 0 = Not at all; 1 = Several days; 2 = More than half of the days; 3 = Nearly every day. Sum scores were used in analyses.

To assess *adherence in terms of attrition* or drop-out rate, the amount of sessions that were actually completed were counted. Each completed session accounted for a 1 and a non-completed session was scored with a 0. Differences between the conditions were then calculated (cf. Ballegooijen et al., [4].

#### Alliance

Alliance formed between the iCBT facilitator and the participant was measured after each session with the Virtual Therapist Alliance Scale (VTAS; [33]; Cronbach’s alpha = 0.92). The VTAS consists of seventeen questions, for example, asking the participant whether the provider could be described as warm. Each item was followed by a 5-point rating scale: 1 = Strongly disagree; 2 = Somewhat disagree; 3 = Neither agree nor disagree; 4 = Somewhat agree; 5 = Strongly agree.

#### Mood

Mood or general mental health was assessed using the Patient Health Questionnaire-9 (PHQ-9; [62]; Cronbach’s alpha = 0.89) prior to (as baseline measure T0), during (T1), and after (T2) completing each of the three sessions as well as post-hoc 3–6 days after the last session (T3). The PHQ-9 consists of nine items preceded by a leading question: “Over the last two weeks, how often have you been bothered by any of the following problems?” An example of a following item is: “Little interest or pleasure in doing things?”. Answering options per item are: “Not at all” (scored 1), “Several days” (scored 2), “More than half of the days” (scored 3), “Every day” (scored 4). The scores from the PHQ-9 were calculated as a summed total and interpreted as follows: 0–4 = None; 5–9 = Mild; 10–14 = Moderate; 15–19 = Moderately severe; 20–36 (max.) = Severe.

#### Satisfaction

Satisfaction with the intervention was measured with the 13 item scale Satisfaction with Therapy and Therapist Scale - Revised (STTS-R; [63]; Cronbach’s alpha = 0.93), with each item followed by the same 1–5 rating scales as in the VTAS. The STTS-R was assessed post-hoc as participants’ overall satisfaction with the therapy 3–6 days after the last session. Example items are whether the participants felt free to express themselves, or whether they would return to the therapy if they needed help. Per session (T0, T1, and T2), participants’ satisfaction with the intervention was simply assessed through a 1-item evaluation rating (1–5 with score 5 being the highest).

### Procedure

Participants were voluntarily recruited from the (first) authors’ university campus through pitches at lectures, posters and flyers with a QR-code, providing information and options to subscribe to 3 individual sessions in a private room at the university. In recruiting participants, the information provided focused on reaching students with mood issues. By clicking the link in e-messages or scanning the QR-code, participants were sent to a form on Qualtrics, where they were asked for their e-mail address and a self-created research ID (consisting of 6–8 digits). They were then asked to read and sign the informed consent form. Participants were free to discontinue their participation in the study at any time. Participants were rewarded with €15 upon completion of the study. For the various steps in the study design, see flowchart in Fig. 5. Data and materials are available on OSF.

### Results

#### Preliminary Results

To examine possible zero order correlations, we correlated the various measures for the dependent variables (see Suppl. Mats., Study 2, Table S3). As expected, sessions correlated within variables, less so for adherence between sessions. Alliance also correlated significantly with Satisfaction, but not with Adherence (per session), even though they were measured quite differently.

Because of the relatively low number of participants, the assumptions for a mixed MANOVA, between-subjects with repeated measures were not met. Non-parametric alternatives, the Wilcoxon or Welch’s tests, were therefore used to analyze the data.

#### Testing Hypotheses

**Fig. 6:**
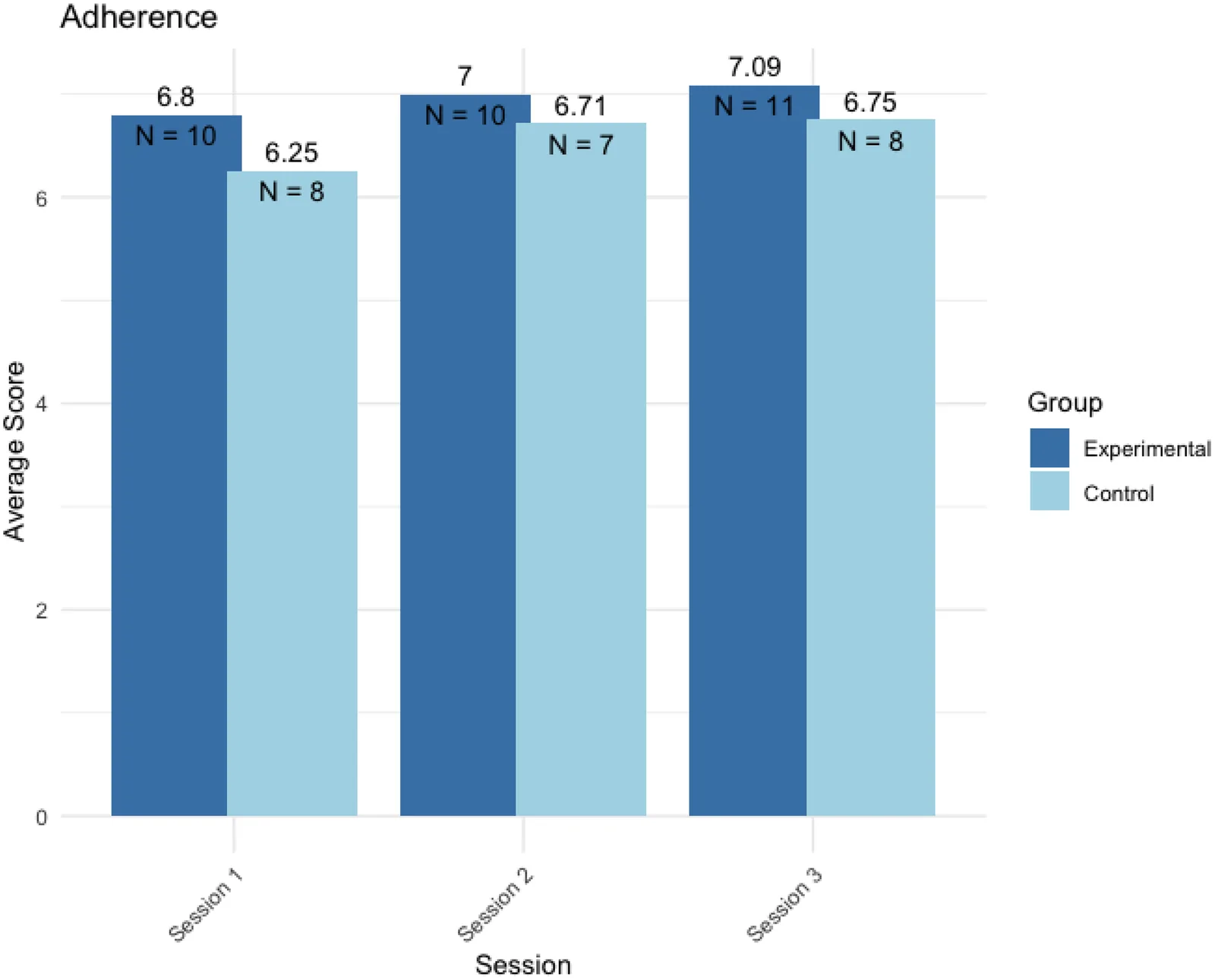
Descriptives for measured adherence per session

To test H1, whether the social robot led to stronger adherence than the screen-based avatar interventions, the descriptives for the measure are shown in Fig. 6 and show a trend in the expected direction. However, results of Welch’s Two Sample Test showed that the difference in adherence between the two conditions over all sessions was not significant (overall *p* = .470), neither per individual session (s1, *p* = .432; s2, *p* = .827; s3, *p* = .744).

Adherence as assessed through number of completed sessions was significant (*p =* .043) with higher attrition in the screen-based control group. The experimental group, with the robot, completed 100% of sessions, whereas the control group completed 87% of all sessions. Two participants from this group dropped out after their first session (i.e., a drop-out rate of 20% (2/10)). Thus, H1 was supported in terms of attrition rate.

**Fig. 7:**
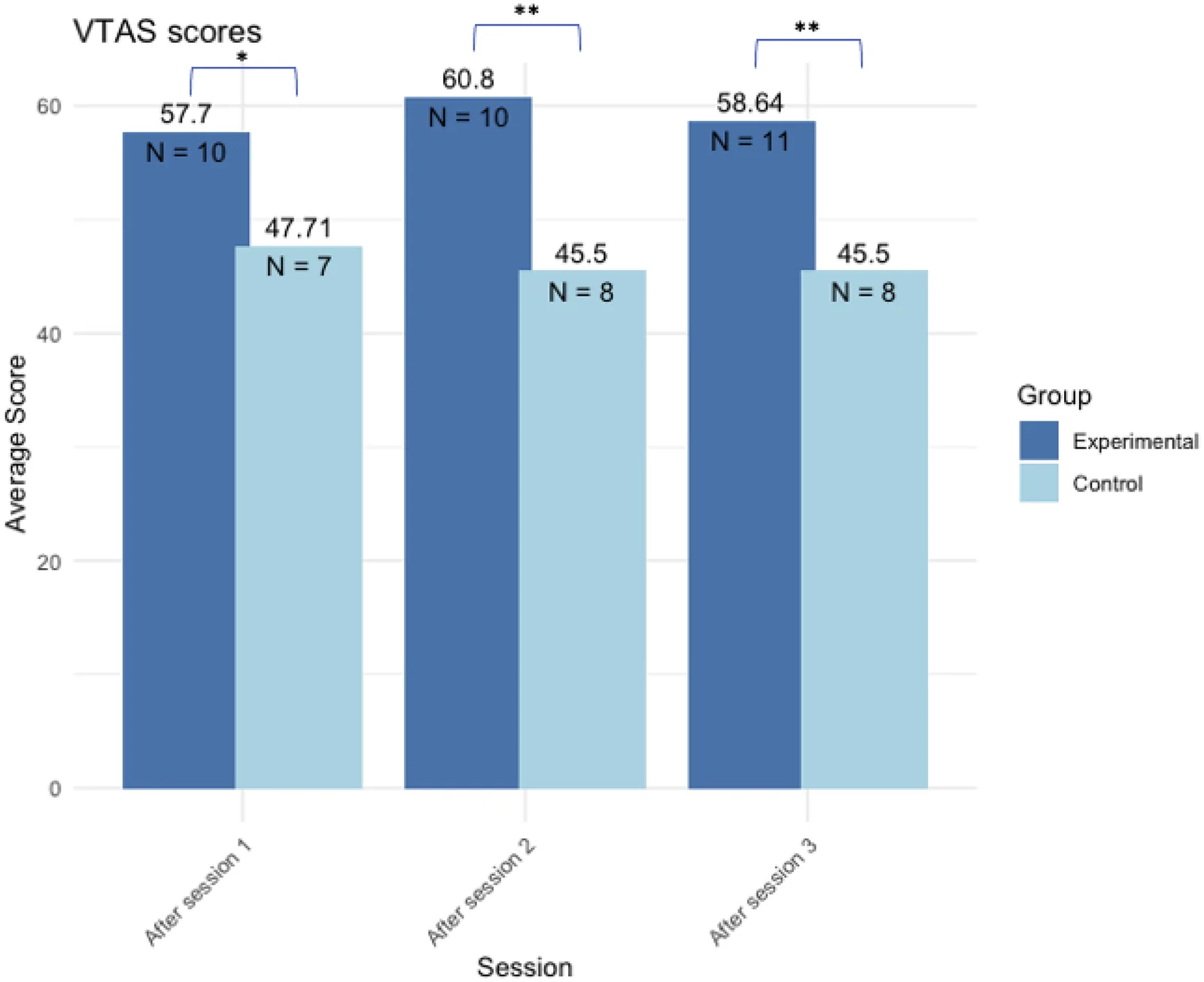
Descriptives for Alliance (VTAS scores) per session

To test H2, whether the social robot led to stronger alliance than the screen-based avatar interventions, the descriptives per session for alliance (measured through VTAS) are shown in Fig. 7 and show the expected direction. The differences between the two conditions were significant according to the results of Welch’s Two Sample Test, for each session (s1: *p* = .030, s2: *p* = .008, s3: *p* = .009), as well as averaged over all (*p* < .001). Results indicated a significantly stronger alliance with the robot than with the avatar, supporting H2.

**Fig. 8:**
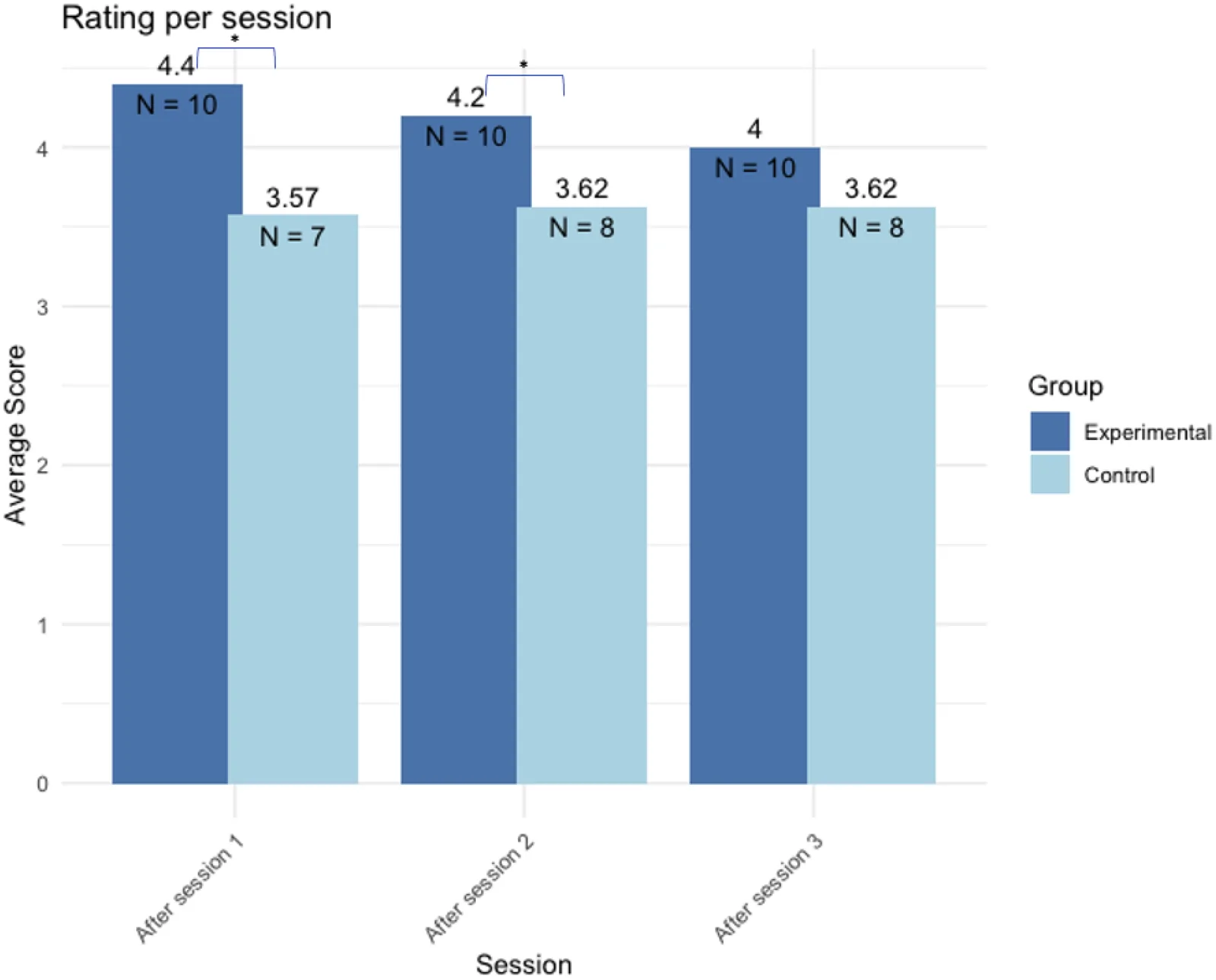
Descriptives for Satisfaction (STTS) per session

Following the same line of thought as H2, we tested whether the social robot led to stronger satisfaction than the screen-based avatar interventions. The descriptives per session for satisfaction (STTS) are shown in Fig. 8 and show the expected direction. The differences between the two conditions were significant according to the results of Welch’s Two Sample Test, for each session (s1: *p* = .030, s2: *p* = .008, s3: *p* = .009), and overall (*p* < .001): Satisfaction with the robot was significantly higher than in the avatar condition.

In analyzing possible effects on participants’ mood states of interventions with the social robot therapist compared to the avatar (cf. H2), the descriptives are reported in Fig. S9 (see Suppl. Mats). Note that a decline of the average score indicates an improvement of participants’ mood over time. Importantly, in the experimental robot-group a relatively low average of *M* = 6.42 at baseline was observed (a score between 5 and 9 corresponds to a mildly depressed mood). Before the second and third sessions, a decline of the average score (and hence an improvement of mood) can be seen, yet, after the third session, the average score increased again. The average severity of the mood issues, however, remained stable over time. The scores of the avatar-control group were higher at baseline, and this difference remained approximately the same throughout. Because the experimental group had on average a somewhat lower baseline score for mood than the control group, the concept of “paired differences” between the groups was used to account for this initial difference (cf. Divine et al., [64].

For the statistical test, only participants who completed all four PHQ-9 measures were included. Hence, in both conditions two participants had to be excluded for this analysis. Applying the non-parametric paired Wilcoxon test, testing “paired differences” between groups [64], no significant differences were found between sessions nor afterwards for the last session (all *ps >* 0.17). The results of the statistical analysis over all (*W* = 518, *p* = 0.6002) indicated that the difference between the two iCBT facilitators had no significant effect on the efficacy of the therapy with regard to participants’ mood.

#### Discussion Study 2

Results of Study 2 showed that an iCBT intervention through a social robot was more effective than through a screen-based avatar in terms of adherence, alliance, and satisfaction with the therapy. Differences in effect on mood were not found nor an improved mood afterwards. Whereas Study 2 had implemented a number of methodological improvements to Study 1, results are still in line.

Strengths of Study 2 are that the intervention implemented an existing iCBT therapy, named MoodPep from Caring Universities [60, 61] whereby the screen-based avatar condition closely resembled current practices and included an avatar that looked the same as the physically embodied robot. The social robot used speech to verbally inform and instruct the participant, strictly following the same protocol as the avatar version. Moreover, all participants had to go to the same location to attend the sessions, that is, also those in the screen-based avatar condition. Hence, conditions were also similar in this respect and thereby mitigated the possible confound in Study 1. In addition, Study 2 applied standardized measurements for the dependent variables following commonly used measurement instruments such as the VTAS [33]. Beyond all adjustments, results still showed that a social robot seems a more suitable replacement for a human therapist in unguided iCBT than a screen-based version with an avatar added.

A limitation of Study 2 was insufficient power due to a smaller sample size than required based on a priori power analysis. Due to practical reasons, we could not collect more data. Nevertheless, significant effects were found in the expected directions. Due to the small sample size, a mediation analysis in PROCESS could not be conducted (as we did in Study 1). While the iCBT intervention with the social robot increased adherence, therapeutic alliance, and satisfaction, no improvements in participants’ mood were found. This absence of mood effects could have two explanations. First, the duration of the study may have been too short for significant mood improvements to emerge. Second, participants already reported a low level of mood-related problems at baseline, resulting in little room for improvement. Together, these factors may explain why no changes in mood were observed despite positive effects on adherence and satisfaction. Whereas the results overall show the potential of a physically embodied social robot to act as facilitator in iCBT, a replication study is warranted with a larger sample size.

### General Discussion

The current research aimed to assess the extent to which a social robot can act as guidance in unguided iCBT interventions. Two experiments compared a social robot as therapist with a text-only website, and a screen-based avatar, each in three subsequent sessions of unguided iCBT. By and large, in both studies, our hypotheses were supported by the results showing that a social robot yields higher levels of therapeutic adherence, alliance, and satisfaction after the interventions with an embodied social robot than the same version of iCBT presented on screen with an avatar (both studies) or without (Study 1). However, no significant improvements were found for participants’ feelings of loneliness (Study 1) or mood (Study 2).

The results showing the highest adherence, alliance, and satisfaction rates for the social robot version are in line with previous findings in different settings (i.e., not iCBT). Research with social robots emphasized that a physical embodiment, in comparison to a virtual embodiment, can yield higher levels of social interaction with the user due to the uniqueness of the social robot’s physical presence, sharing the same space as the user [41, 43, 44, 65, 66] and instructions are followed more closely [14]. Lee and colleagues (2015) explained that by being physically present, the social robot is perceived more positively and receives more attention, while others argue that physically embodied social robots more easily elicit feelings of connectedness or a socio-affective bond [12, 16, 41, 42], which is essential in a therapeutic setting.

In contrast to previous findings however, the social avatar version in Study 1 did not achieve better alliance than the text-only approach. Earlier research indicated that a social avatar was significantly better than a text-only alternative on the level of alliance ([37]; Hein et al., 2018; Provoost et al., 2017). Further research could examine more thoroughly the functions, affordances, and design features of social avatars in iCBT (e.g., in speech, gestures and behavior) and their role in adherence and alliance, also in comparison to a social robot.

Perhaps the most promising results of our studies relate to increased therapeutic alliance due to the physical embodiment of a social robot as therapist. That is, the robotic iCBT facilitator provided better results through the therapy sessions than common iCBT with an avatar in terms of alliance to the therapeutic facilitator and therapy. The embodied, physical presence of a social robot elicited feelings of alliance more so than the screen-based social avatar in the context of unguided iCBT. It might well be that such physical embodiment elicits feelings of being in a social interaction with related social behavior as-if in a human-human interaction, including human responsiveness to related social cues. An embodied social robot present in the same physical space in 3D as the participant and signaling humanlike cues is probably more likely to trigger feelings of social connection than just a flat screen. This is in line with human-robot interaction studies in related areas showing the ease of more intuitive social interaction and relationship formation with a social robot (e.g., [12, 13, 41]; Lee et al., 2015; [14]) and recent theorizing to explain human-robot affective bonding in a multidisciplinary framework [42]. In addition, participants were also more satisfied with the therapeutic intervention by the robot than the avatar after each session, as well as after all sessions were completed.

Importantly, adherence was mediated by therapeutic alliance in case of the embodied robotic therapist as shown in Study 1, explaining the higher levels of adherence to the robot-guided treatment. Hence, our empirical evidence supports that the formation of an alliance between client and therapist results in a higher level of adherence, and thus clients less likely to drop-out of therapy [53], even when the therapist is just a social robot. Likewise, our study showed that participants who experienced higher levels of alliance with the robot-therapist also performed the exercises more often, resulting in higher adherence levels for participants interacting with the social robot. The larger drop-out rates in the text-only condition (Study 1) and the avatar condition (Study 2) can also be seen as an indicator for stronger adherence when a social robot is included in the sessions. Thus, a social robot seems to have potential to overcome adherence issues in unguided iCBT, while therapy adherence is crucial to the therapy’s effectiveness [4, 11]. Interestingly, given the increased therapeutic alliance induced by a social robot supporting the therapeutic intervention, we may wonder whether a physically embodied social robot acting as iCBT facilitator in fact acts as ‘guidance’ (despite being considered ‘unguided’ treatment because of the absence of a human therapist). This might be further explored in future research.

Not having found significant improvements for the participants’ loneliness (in Study 1) or mental state as assessed through the PHQ-9 (in Study 2) might be due to the relatively mentally healthy participants (cf. baseline measure) and perhaps the short duration of the intervention (i.e., just three sessions). The baseline measurements indicated that on average and before the trial, the participants in the experimental group had fewer mood issues than in the control group. In this respect, the randomization procedure did not work as expected and the relatively low baseline levels leave little room for mood improvements. Nevertheless, given the increased adherence, alliance, and satisfaction, results do highlight the potential of social robots in enhancing effectiveness of unguided iCBT. In future research, more prolonged interventions should be tested with a larger sample of participants who do show some degree of mood issues. It would then also be interesting to compare a human-guided therapeutic intervention with a social robot guided version, preferably assisted by Artificial Intelligence (AI) or a Large Language Model (LLM) to allow for a more natural interaction.

An interesting question for future research is whether implementation of LLMs and GenAI in social robots and chatbots, improving communication capabilities, might change the importance of the physical embodiment of social robots in establishing (therapeutic) alliance and improving adherence. We argue that more fluent and naturalistic conversations through LLMs will increase effectiveness of *both* modalities and it remains to be seen for whom and under which circumstances, one or the other might appear most optimal. In all, we believe that the added value of the physical embodiment of social robots, compared to screen-based applications, might depend in part on the specific target group and goal or task for which it is used. We are still at the beginning of examining the potential of social robots for therapeutic interventions.

Limitations of Study 1 were adequately addressed in Study 2 and both studies complement each other in their strengths and weaknesses. For example, the possible confound in Study 1 that participants had to put more effort in going to a location for the intervention with the robot was overcome in Study 2 where all participants had to go to the same location. Thus, even though just putting more effort into acquiring therapy could already be sufficient to achieve better results, which would then have equally applied to the avatar condition, was ruled out. Because Study 2 suffered from insufficient power, no mediation analysis could be carried out, whereas Study 1 had sufficient power to do so. Moreover, despite the relatively low number of participants, results of both studies are in the same direction. Thus, both studies strengthen each other in the validity of results.

Generalizability of results is limited, however, given the student population, being young adults. Future research could try to include actual clients from waitlists after extensive ethical considerations, guarding privacy issues, and evaluations of design, materials and procedure in close cooperation with relevant stakeholders.

In concluding, the findings of this research are promising in showing that a humanlike robotic iCBT facilitator of unguided therapy was more effective than comparable screen-based versions in terms of therapy adherence, alliance to the therapeutic facilitator, and being satisfied with the intervention. These results merit further, larger-scale studies to determine the full extent of the benefits of robot-assisted iCBT. Future research could explore making robots more appropriately tailored in their interactions, for example, by using Large Language Models and AI, and investigate the impact of robot-assisted therapy on participants with various mood issues or depression severity levels. This research contributes to understanding the effectiveness of alternative types of unguided therapy facilitators, which is highly needed in view of current and future shortage in healthcare professionals. Social robots appear to be a most suitable alternative for providing unguided iCBT, without the presence of a human therapist. People in need of therapy may want someone around even if that someone turns out not to be a real human.

## Electronic Supplementary Material

Below is the link to the electronic supplementary material.

Supplementary Material 1

## Data Availability

Data are available on OSF: https://osf.io/f2huk. Supplementary Materials are available via the link above, on p. 15.
