# Supplementary material for: Examining the Potential of Social Robots to Increase Adherence in Internet-based CBT
Source: Int J Soc Robot. 2026 Jul 13;18(6):83. doi: 10.1007/s12369-026-01417-8 (PMC13364896; doi:10.1007/s12369-026-01417-8)
Supplement: Supplementary file 1 — Supplementary Material 1 [file 12369_2026_1417_MOESM1_ESM.docx]

**Supplementary Materials (Suppl. Mats.) for**

**“Examining the Potential of Social Robots to Increase Adherence in Internet-based CBT”**

**Table S1**

*Zero-order Correlations between Variables in Study 1.*

| Variables | 1 | 2 | 3 | 4 | 5 | 6 |
| --- | --- | --- | --- | --- | --- | --- |
| 1.Gender | - |  |  |  |  |  |
| 2.Age | .00 | - |  |  |  |  |
| 3.Adherence | -.235 | .060 | - |  |  |  |
| 4.Alliance | -.375* | -.119 | .703** | - |  |  |
| 5.Loneliness_T1 | -.195 | -.215 | .050 | .159 | - |  |
| 6.Loneliness_T2 | -.195 | -.207 | .125 | .261 | .771*** | - |

*Notes.* For gender, 0 = male, 1 = female. * *p* < .05, ** *p* < .01, ***, *p* < .001

**Mediation Analyses Study 1**

The results of comparing the avatar condition to the text-based condition showed no significant direct relationship between the type of unguided facilitator and adherence (*B* = .55, *SE* = .37, *p* = .151) (Figure S3.a). Furthermore, no significant relationships were found for these two conditions on the feeling of alliance (*B* = 0.24, *SE* = .41, *p* = .554). Moreover, the indirect relationship for the type of unguided therapeutic facilitator on adherence through alliance was not significant. Although stronger feelings of alliance did result in significantly higher levels of adherence (*B* = .44, *p* = .011), no mediation of alliance could be established for the social avatar as compared to the text-based website.

**Figure S3.a.** *Mediation analysis (text-based website vs. social avatar)*

Alliance

Adherence

b = .44^*^; *SE* = .16

c’ = .55^ns^; *SE* = .37

c = .65^ns^; *SE* = .41

a = .24^ns^; *SE* = .37

Social avatar

vs

Text-based website

*Note.* Mediation analysis predicting indirect effects between the unguided iCBT facilitator (text-based website vs. social avatar) and Adherence through Alliance. Unstandardized path coefficients, **p <* 0.05*, ** p <* 0.01*, *** p <* 0.001*.*

Including the text-based website and the social robot, the results demonstrated that participants working with the robot showed significantly stronger Adherence (*B* = 1.72, *p* < .001, see Figure S3.b) than those working with the text-only version. Furthermore, the type of unguided facilitator was significantly and positively related to alliance (Figure S3.b; *B* = 1.70, *p* < .001), resulting in a stronger relationship for adherence (*B* = .44, *p* = .011). Testing the indirect mediation path to adherence via alliance, result of the Sobel test was significant (*z =* 2.29, *p* = .022) and the indirect path to adherence via alliance significantly differed from zero according to the Confidence Interval (point estimate = .73, 95% *CI* [.16, 1.60]). Thus, the higher level of adherence in the social robot condition, as compared to the text-only condition, is mediated by feelings of alliance.

**Figure S3.b.** *Mediation analysis (text-based website vs. social robot)*

Social robot

vs

Text-based website

Alliance

Adherence

c’ = 1.72^***^; *SE* = .41

c = 2.46^***^; *SE* = .41

b = .44^*^; *SE* = .16

a = 1.70^***^; *SE* = .41

*Note.* Mediation analysis predicting indirect effects for the type of unguided facilitator (text-based website vs. social robot) and adherence through alliance. Unstandardized path coefficients*, * p <* 0.05*, ** p* < 0.01*, *** p <* 0.001*.*

**Table S2**: **Loneliness scores between the different conditions in Study 1**

| *condition* | ***M* (SD) Before** | ***M* (SD) After** |
| --- | --- | --- |
| unguided iCBT provided by a text-based website | *M* = 3.27; *SD* = 2.01 | *M* = 2.67; *SD* = 1.46 |
| unguided iCBT provided an avatar on website | *M* = 3.46; *SD* = 1.66 | *M* = 1.85; *SD* = 1.46 |
| unguided iCBT provided by a robot | *M* = 3.46; *SD* = 1.56 | *M* = 3.31; *SD* = 1.44 |

*Note. Min. score is 1, max. score = 6*

The loneliness scores between the different conditions did not significantly differ before (*F*_(2,34)_ = 0.046, *p* = .955) and after the experiment (*F*_(2,32)_ = 0.45, *p* = .639).

**Suppl. Mats to Study 2**

**Table S3.** *Zero-order Correlations between Variables in Study 2.*

| **Variable** | **Adherence session 1** | **Adherence session 2** | **Adherence session 3** | **Alliance score after session 1** | **Alliance score after session 2** | **Alliance score after session 3** | **PHQ-9 score before the first session** | **PHQ-9 score before the second session** | **PHQ-9 score before the third session** | **PHQ-9 score after the third session** | **STSSR** |
| --- | --- | --- | --- | --- | --- | --- | --- | --- | --- | --- | --- |
| Adherence session 1 | 1.00 | 0.40 | **0.50*** | 0.13 | 0.12 | 0.39 | -0.11 | -0.02 | -0.02 | -0.16 | 0.26 |
| Adherence session 2 |  | 1.00 | 0.71 | 0.13 | 0.39 | 0.39 | 0.13 | 0.04 | -0.05 | 0.28 | 0.39 |
| Adherence session 3 |  |  | 1.00 | 0.00 | 0.30 | 0.33 | 0.10 | 0.13 | -0.04 | 0.07 | 0.41 |
| Alliance score after session 1 |  |  |  | 1.00 | **0.78*** | **0.69*** | -0.25 | -0.58* | -0.34 | -0.37 | **0.68*** |
| Alliance score after session 2 |  |  |  |  | 1.00 | **0.83*** | 0.02 | -0.36 | -0.24 | -0.04 | **0.79*** |
| Alliance score after session 3 |  |  |  |  |  | 1.00 | -0.22 | -0.39 | -0.27 | -0.28 | **0.83*** |
| PHQ-9 score before the first session |  |  |  |  |  |  | 1.00 | **0.69*** | 0.39 | **0.61*** | -0.19 |
| PHQ-9 score before the second session |  |  |  |  |  |  |  | 1.00 | **0.49*** | **0.70*** | -0.26 |
| PHQ-9 score before the third session |  |  |  |  |  |  |  |  | 1.00 | **0.44** | -0.42 |
| PHQ-9 score after the third session |  |  |  |  |  |  |  |  |  | 1.00 | -0.05 |
| STSSR |  |  |  |  |  |  |  |  |  |  | 1.00 |

***Note. **** *Correlation is significant at the 0.05 level (2-tailed);* ******** *Correlation is significant at the 0.01 level (2-tailed).*

***Figure S9:*** *PHQ-9 scores of participants who completed the PHQ-9 for all four measurement times.*


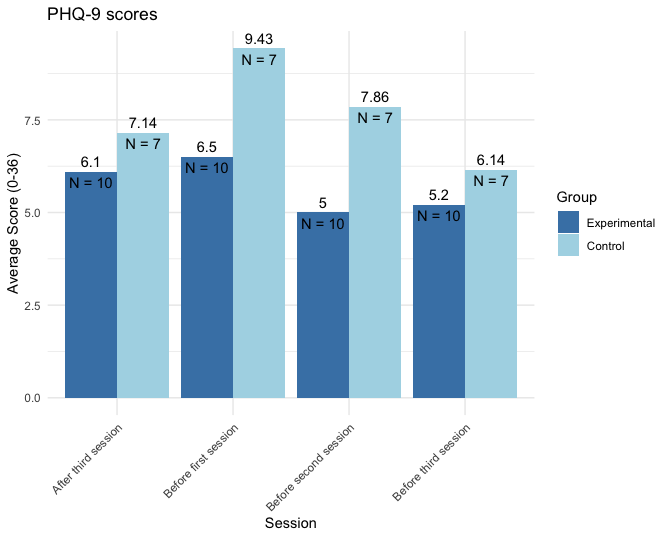


Note. The dark blue bars represent averages of the experimental group per session (robot), the light blue bars represent the control group (avatar). The first couple of bars represents the average of the scores of the PHQ-9 at the final measure moment, after the treatment; the second represents the baseline measure; the third and the fourth couple of bars represent the in-between measures at the start of sessions. The ‘N’ within each bar represents the number of participants that contributed towards the average, which can be seen on top of every bar.
